# Vutiglabridin Modulates Paraoxonase 1 and Ameliorates Diet-Induced Obesity in Hyperlipidemic Mice

**DOI:** 10.3390/biom13040687

**Published:** 2023-04-18

**Authors:** Dawoud Sulaiman, Leo Sungwong Choi, Hyeong Min Lee, Jaejin Shin, Dong Hwan Kim, Keun Woo Lee, Pierre Eftekhari, Angélique Quartier, Hyung Soon Park, Srinivasa T. Reddy

**Affiliations:** 1 Department of Medicine, Division of Cardiology, David Geffen School of Medicine, University of CA Los Angeles, Los Angeles, CA 90095, USA; 2 Glaceum Incorporation, Suwon 16675, Republic of Koreajaejin@glaceum.com (J.S.);; 3 Department of Bio & Medical Big Data, Division of Life Science, Research Institute of Natural Science, Gyeongsang National University, Jinju 52828, Republic of Korea; 4 Inoviem Scientific, 67400 Illkirch-Graffenstaden, France

**Keywords:** obesity, hyperlipidemia, PON1, vutiglabridin

## Abstract

Vutiglabridin is a clinical-stage synthetic small molecule that is being developed for the treatment of obesity and its target proteins have not been fully identified. Paraoxonase-1 (PON1) is an HDL-associated plasma enzyme that hydrolyzes diverse substrates including oxidized low-density lipoprotein (LDL). Furthermore, PON1 harbors anti-inflammatory and antioxidant capacities and has been implicated as a potential therapeutic target for treating various metabolic diseases. In this study, we performed a non-biased target deconvolution of vutiglabridin using Nematic Protein Organisation Technique (NPOT) and identified PON1 as an interacting protein. We examined this interaction in detail and demonstrate that vutiglabridin binds to PON1 with high affinity and protects PON1 against oxidative damage. Vutiglabridin treatment significantly increased plasma PON1 levels and enzyme activity but not PON1 mRNA in wild-type C57BL/6J mice, suggesting that vutiglabridin modulates PON1 post-transcriptionally. We further investigated the effects of vutiglabridin in obese and hyperlipidemic LDLR^−/−^ mice and found that it significantly increases plasma PON1 levels, while decreasing body weight, total fat mass, and plasma cholesterol levels. Overall, our results demonstrate that PON1 is a direct, interacting target of vutiglabridin, and that the modulation of PON1 by vutiglabridin may provide benefits for the treatment of hyperlipidemia and obesity.

## 1. Introduction

Obesity is the leading cause of the metabolic syndrome comprising insulin resistance, hypertension, and hyperlipidemia, all of which are risk factors for the development of atherosclerosis and cardiovascular diseases [1,2]. Intake of high-fat and high-sugar Western diet (WD) is considered a major contributor to the growing rate of obesity worldwide [3]. Excess intake of sugar and fat leads to whole-body insulin resistance and an increased level of plasma free fatty acids [4], which in turn induces lipid peroxidation and oxidative modification of low-density lipoprotein (LDL) leading to increased levels of oxidized LDL (oxLDL) [5]. High levels of oxLDL coupled with elevated levels of phospholipid and free fatty acids present in obese conditions are well-known triggers of atherogenesis [6]. The oxLDLs are scavenged and taken up by macrophages that transform into foam cells, which later become fatty streaks in the endothelium and form atheromatous plaques [7]. Thus, WD-induced obesity is characterized by not only an increase in total body weight and fat mass, but also an increase in plasma lipids, especially LDL and oxLDL. The LDL receptor (LDLR) in the liver mediates approximately 70% of the endocytosis of LDL for its clearance [8]. Moreover, LDLR-knockout (LDLR^−/−^) mice fed a WD display a significant increase in total body weight, fat mass, and increased plasma lipids [9,10]. Consequently, LDLR^−/−^ mice serve as a useful and representative model of obesity and obesity induced hyperlipidemia.

Paraoxonase 1 (PON1) is an extensively studied plasma protein that confers antioxidant properties of high-density lipoprotein (HDL) [11]. PON1 is synthesized in the liver and secreted into the bloodstream, where it binds and associates with HDL [12,13]. As a hydrolytic enzyme, PON1 possesses lactonase, arylesterase, and paraoxonase activities [14]. While the exact mechanism of the antioxidative function of PON1 is not known [15], PON1 was found to protect HDL and LDL from oxidation by reducing their peroxide content [16]. Peroxidase activities of PON1 include hydrolysis of hydrogen peroxide, lipid peroxides, especially cholesteryl linoleate hydroperoxides, and peroxide phospholipids in oxLDL [16,17]. As a result, PON1 has been linked to various metabolic disorders, including atherosclerosis, type 2 diabetes, non-alcoholic fatty liver disease, and obesity [7]. In obese subjects, PON1 activity was significantly lower than in non-obese subjects, while lipid hydroperoxides in HDL and LDL were higher and HDL-C reduced. [18,19]. Although there are certain pharmacological drugs such as statins that lower LDL level, and dietary factors such as polyphenols that increase PON1 expression or activity, to date, there have been no clinically developed drugs that target and directly modulate PON1 [20].

Vutiglabridin (official INN name; previously known as HSG4112) is an orally administered small molecule that is being developed for the treatment of obesity. It has completed Phase I clinical studies on safety and pharmacokinetics in both lean and obese subjects (NCT04732988, NCT04733001, NCT04703764), and a Phase II study on efficacy in obese subjects is currently ongoing (NCT05197556). Vutiglabridin is a synthetic derivative of glabridin [21], a natural compound and the key chemical component of licorice, which is known for not only its antioxidative and anti-inflammatory functions but also a robust bodyweight reducing capacity [22]. Vutiglabridin was found to be the most efficacious compound selected from an in vivo phenotypic screening method, where various synthetic derivatives of glabridin were administered directly to high-fat-diet-induced obese mice [21]. Given this phenotypic screening discovery method, the exact target protein(s) of vutiglabridin are not yet known.

Nematic Protein Organisation Technique (NPOT) is a label-free proprietary technology offered by INOVIEM Scientific and is used to isolate and identify specific macromolecular scaffolds that form when the compound is incubated directly with the animal tissue of interest. This technique is based on the Kirkwood–Buff molecular crowding and aggregation theory [23,24], wherein proteins that interact directly with the compound or that interact with target proteins form nematic crystals and grow into scaffolds under certain liquid phase. Those scaffolds are then isolated for proteomic identification. This technique has been employed previously to successfully identify the target protein of Trolox-based small molecule KH176 and various other small molecules as well [25,26,27].

In this study, we identify PON1 as a direct interacting protein of vutiglabridin via NPOT analysis and show that vutiglabridin increases plasma level of PON1 in both wild-type and obese/hyperlipidemic mice. Our results suggest, for the first time, that the effects of vutiglabridin against obesity and hyperlipidemia are, in part, mediated by the modulation of PON1 levels and activity.

## 2. Materials and Methods

### 2.1. Nematic Protein Organisation Technique (NPOT^®^), Heteroassembly Isolation, and Proteomics

The protocol generally follows a previously described method [27]. Briefly, liver from C57BL/6J wild-type (WT) or high-fat (60% kcal fat) diet-induced obese (DIO) mice were homogenized at 4 °C in the absence of any detergent, reducing agent, or protease/phosphatase inhibitors, and vutiglabridin 1 µM was added. A differential microdialysis system was used, based on a transitory pH gradient (pH 5–10) where the macromolecules (protein groups) migrate in the liquid phase to their mean molecular zwitterion positions, to separate the macromolecular assemblies. The migrating macromolecules grew gradually from nematic crystals to macromolecular heteroassemblies due to the molecular interactions between the compound and its target proteins. The heteroassemblies were then trapped in mineral oil and isolated for LC-MS/MS analysis. All dilutions and washes were performed in standard Hepes saline buffer solution (HBSS) with equal osmolality, trace elements, vitamins, and salts in concentrations as close as possible to those of the interstitial medium or cellular cytoplasm (Inoviem Scientific proprietary buffer).

For LC-MS/MS analysis, the heteroassemblies were solubilized directly in 10 μL of 2D buffer (7 M Urea, 2 M Thiourea, 4% CHAPS, 20 mM DTT, 1 mM PMSF), and proteins were precipitated in acetate buffer by centrifugation for 20 min at 7500× *g*. Pellets were digested for 1 h with Trypsin Gold (Promega, Madison, WI, USA) at 37 °C. Trypsin Gold was resuspended at 1 μg/μL in 50 mM acetic acid, and then diluted in 40 mM NH_4_HCO_3_ to 20 μg/mL. The samples were dried in SpeedVac at room temperature. Peptides were purified and concentrated using ZipTip pipette tips (Millipore Corporation, Burlington, MA, USA). Mass spectrometry analysis was performed for 1 h in a timsTOF Pro machine (Bruker, Billerica, MA, USA). Proteins were identified using Mascot software (version 2.6.1).

### 2.2. Bioinformatics Analysis

Based on the proteins identified through NPOT, a Gene Ontology (GO) search in an unbiased manner was performed to explore the top five relevant cellular components (CC), molecular functions (MF), and biological processes (BP) using g:Profiler software. Statistical analysis of GO was performed using g:SCS threshold with a *p*-value < 0.05. To evaluate the proteins involved with the pathogenesis of hyperlipidemia, a protein–protein interaction network was built with the protein–protein interactome information from the STRING 9.1 public database; proteins involved in steroid metabolic process, phospholipid metabolic process, and lipid transport were selected.

### 2.3. In Silico Molecular Docking Study

Molecular docking calculations between the compounds and the PON1 binding sites were carried out by Genetic Optimization for Ligand Docking (GOLD v5.2.2, The Cambridge Crystallographic Data Centre, Cambridge, UK). GOLD is an automated docking program to predict the binding mode of the ligand by genetic algorithm [28,29]. The crystal structure of human PON1 (PDB ID: 1V04) was obtained from RCSB Protein Data Bank (http://www.rcsb.org (accessed on 19 January 2021)). The 3D structures of compounds were prepared using Discovery Studio (DS) 2018 (BIOVIA, San Diego, CA, USA). The geometry of the compounds was optimized by energy minimization through Minimize Ligands tool in DS. All the active site residues within 10 Å radius sphere of the center were included for the calculation. The number of docking runs was set to 50 for each hit compound. All other parameters were set as default. Docked conformations of hit compounds were filtered based on the consistency with their mapped poses to the pharmacophore queries obtained from the database screening.

### 2.4. Recombinant PON1 Lactonase Activity Assay

The protocol was generally adapted from a previous study [30]. Linoleic acid, 5,5′-dithiobis (2-nitrobenzoic acid; DTNB), 2-hydroxyquinoline, and glabridin were purchased from Sigma-Aldrich (St. Louis, MO, USA). TBBL (5-thiobutyl butyrolactone) was provided by Glaceum Inc. (Suwon, Republic of Korea). Recombinant PON1 (rePON1) as the purified recombinant human Paraoxonase 1 mammalian cell lysate protein expressed in CHO-K1 cells with a carboxy-terminal hexahistidine tag was purchased from Thermo Fisher Science (Catalog Number RP-75678; Waltham, MA, USA). Oxidized linoleic acid (OX-LA) was prepared by incubating linoleic acid (20 mM stock solution in DMSO diluted to 4 mM in phosphate-buffered saline, pH 7.4) with CuSO_4_ (200 μM stock solution in double-distilled water diluted to 5 μM) for 20 h at 37 °C. OX-LA was extracted from the aqueous solution by an organic solvent (ethyl acetate). The organic layer was evaporated under nitrogen and analyzed by LC−MS/MS. OX-LA was dissolved in DMSO and stored at −20 °C.

Glabridin (10 mM), (S)-vutiglabridin, (R)-vutiglabridin, and vutiglabridin (1, 3, 10 mM each) were incubated with 0.2 μL of rePON1 for 30 min at room temperature (RT). Then, 0.5 μL of OX-LA (1 mM) was added and the resulting mixture was incubated for another 2 h at RT in Tris−HCl buffer (50 mM, pH 8) containing 1 mM CaCl_2_. A 25 μL aliquot of DTNB (0.5 mM in Tris−HCl buffer) was placed in a visible (vis) enzyme-linked immunosorbent assay (ELISA) microplate well (386 well). Then, 10 μL of the rePON1 sample described above (10 U/mL) and 20 μL of 5-thiobutyl butyrolactone (TBBL; 2 mM in Tris−HCl buffer) were added. The catalytic activity of rePON1 was measured spectrophotometrically (SpetraMax M2, Molecular Devices, San Jose, CA, USA) at 420 nm every 2 min for 1 h.

### 2.5. Recombinant PON1 Lactonase Kinetic Assay

The recombinant PON1, OX-LA, DTNB, TBBL and assay buffer used the same materials as the above PON1 lactonase activity assay. rePON1 (2.5 µL) and vutiglabridin (30 µM) were incubated for 1 h at RT. Then, 0.5 μL of OX-LA (1 mM) was added and the resulting mixture was incubated for another 1 h at RT in Tris−HCl buffer (50 mM, pH 8) containing 1 mM CaCl2. Next, 25 μL of DTNB (0.5 mM in Tris−HCl buffer) was placed in a visible (vis) enzyme-linked immunosorbent assay (ELISA) microplate well (386 well). Then, 15 μL of the rePON1 sample described above (10 U/mL) and 20 μL of TBBL (4 mM in Tris−HCl buffer) were added. The catalytic activity of rePON1 was measured spectrophotometrically (SpetraMax M2, Molecular Devices, San Jose, CA, USA) at 420 nm every 2 min for 30 min. K_m_ and V_max_ of the kinetic graph obtained above were extracted through the iFIT program [30,31].

### 2.6. Surface Plasmon Resonance (SPR) Analysis

The glycosylation status of the full-length human recombinant PON1 protein (Origene #TP310356, produced in HEK293T, C-Myk/DDK tagged; Origene, Rockville, MD, USA) was evaluated by digestion of N-glycosylated moieties with the Peptide-N-Glycosidase F (NEB #P0704S, New England Biolabs, Ipswich, MA, USA).

A streptavidin-biotin capture SPR strategy was used: tagged rePON1 protein was captured with a specific biotinylated antibody (mouse anti-DDK/FLAG tag antibody, Origene #TA150015; Origene, Rockville, MD, USA) that was previously captured to the sensor chip surface covered by streptavidin. Antibody was diluted in running buffer (HBS-EP, 10 mM Hepes, 150 mM NaCl, 3 mM EDTA, 0.05% *v*/*v* surfactant P20) and injected over the chip surface until the desired immobilization level was reached. To perform binding assays in kinetics analysis mode the proteins were immobilized at low immobilization levels. In addition, a reference surface was prepared by injecting biotin. Vutiglabridin was dissolved in DMSO at 20 mg/mL and this stock solution was diluted at 10 µM in 10 mM HCl and distilled water. Further serial dilutions were performed in HBS-EP. Vutiglabridin at concentrations of 0.125 μM, 0.25 μM, 0.5 μM, and 1 μM were injected over the flow cells at a flow rate equal to 10 μL/h, with KINJET %pos/40/60 as analyte position, volume, and dissociation time, respectively. The sensor chip surface was regenerated with 10 mM HCl. The specific binding was calculated using BIAeval 3 software (Biacore, GE Healthcare, Chicago, IL, USA) with the postulate of 1:1 ligand/analyte interaction. Based on the Chi^2^ value in regard to the R_max_ (less than 10%) and the difference interval ±2 Response Unit (RU) theoretic fitting and experimental results, the best fitting algorithm between Langmuir or drifting base line was applied.

### 2.7. Mice and Diets

C57BL/6J background WT and LDLR^−/−^ mice were purchased from Jackson Laboratories (Bar Harbor, ME, USA). Both male and female mice were utilized for these experiments and ranged from 8 to 12 weeks of age, as further specified in each experimental design as noted in the respective figure legends. Based on the experimental design, mice were fed a standard mouse chow diet (Ralston Purina, St. Louis, MO, USA) or a Western diet (WD; Teklad, Harlan, catalog# TD88137) with and without vutiglabridin (provided by Glaceum Inc., Suwon, Republic of Korea), which was mixed with the diet at a dose of 100 mg/kg. All mice were weighed both prior to start of the experiment and every subsequent week until termination. Mice were fasted overnight prior to blood and tissue collection.

### 2.8. Plasma Lipids and Glucose Measurement

Plasma lipids, including triglycerides, total cholesterol, high-density lipoprotein (HDL), and unesterified cholesterol, as well as glucose levels were determined using enzymatic colorimetric assays [32], with final concentrations expressed as mg/dL.

### 2.9. Body Composition Measurement

Body composition, specifically total body fat and lean mass, was determined using quantitative nuclear magnetic resonance (NMR) via the Bruker MiniSpec utilizing software from Echo Medical Systems (Houston, TX, USA). The percent of total body fat and lean (muscle) mass was determined by dividing the total body fat/lean mass by total body weight, taken just prior to NMR, and then multiplying by 100.

### 2.10. Immunoblotting

For immunoblotting, 50 μg of protein from plasma and liver lysates was loaded on 4–20% Mini-PROTEAN TGX Stain-Free protein gels (Bio-Rad, Hercules, CA, USA) and transferred to nitrocellulose membranes. The membranes were blocked for 1 h at room temperature in Tris-buffered Saline with 0.1% Tween 20 (TBST) containing 5% (*w*/*v*) milk. The following primary antibodies were used at the specified dilutions: PON1 (catalog# MAB4926, R&D Systems, Minneapolis, MN, USA) at 1:1000 and Transferrin (catalog# S.C.-373785, Santa Cruz Biotech, Dallas, TX, USA) at 1:5000. Primary antibodies were diluted in TBST containing 5% milk at 4 °C overnight, followed by incubation with appropriate secondary antibody (1:5000 dilution) coupled to HRP for 1 h. Protein was detected using Immobilon Western Chemiluminescent HRP Substrate (Millipore Sigma, Burlington, MA, USA). Western blot images were quantified using ImageJ software.

### 2.11. Lactonase, Arylesterase, and Paraoxonase Assays

Plasma and liver homogenates (20 mg) were utilized for the lactonase and arylesterase assays. The protocol followed previously reported method for the lactonase assay [33] and the arylesterase assay of plasma [34]. Briefly, the lactonase assay was performed with dihydrocumarin (Catalog# 104809-5G, Sigma-Aldrich, St. Louis, MO, USA) and the arylesterase assay was performed with phenyl acetate (catalog# P-2396, Sigma-Aldrich, St. Louis, MO, USA), and 96-well flat bottom UV plate (catalog#: 3635, Costar, Washington, DC, USA) was used to quantify the hydrolyzed phenol product absorbance at 270 nm for both assays. Plasma was utilized for the paraoxonase assay, which utilized diethyl p-nitrophenyl phosphate (paraoxon) as the substrate and measuring the increase in the absorbance at 405 nm due to the formation of 4-nitrophenol over a period of 12 min (at 20 s intervals) as previously reported [35].

### 2.12. Statistical Analysis

Student’s *t*-tests were carried out between all studies with two groups. Data from experiments with two or more groups were compared using a one-way ANOVA model. After a significant overall group effect was established, Tukey’s pairwise comparisons were carried out to determine specific group to group differences. Experiments were repeated two to three time to ensure that the results were consistent and reproducible. All data are represented as mean ± SEM. A *p*-value < 0.05 was considered statistically significant.

## 3. Results

### 3.1. Identification of Potential Target Proteins of Vutiglabridin through NPOT Assay

**Figure 1:**
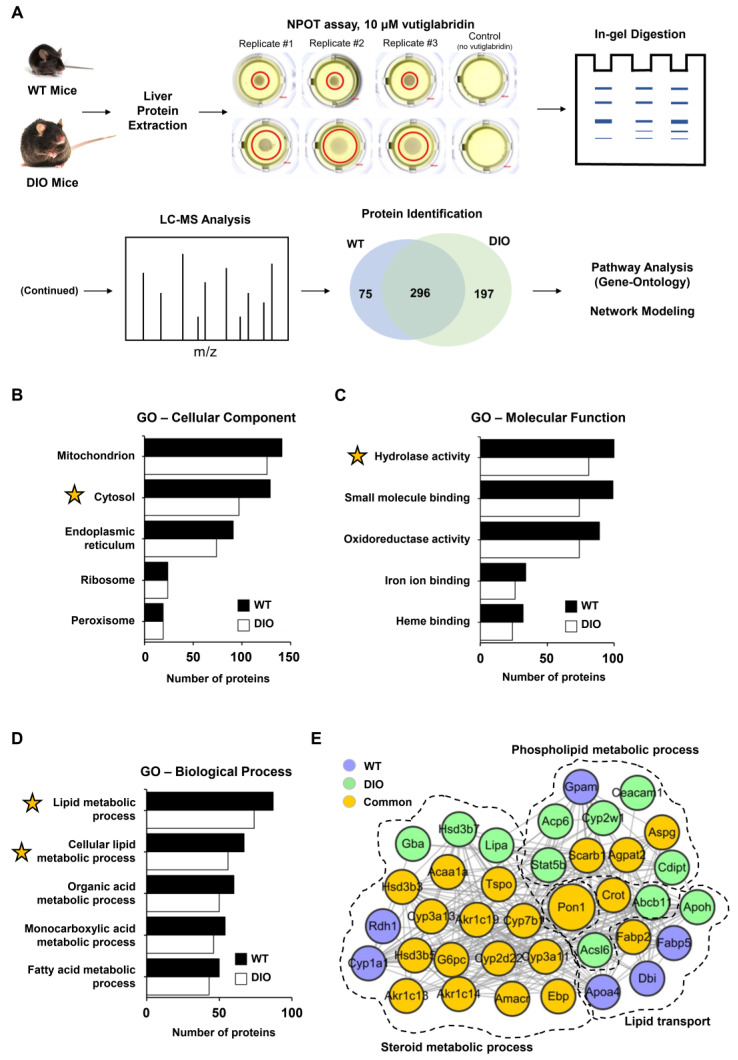
PON1 is identified as a potential target of vutiglabridin through NPOT assay. (**A**) Schematic representation of the Nematic Protein Organization Technique (NPOT) analysis of vutiglabridin on protein homogenates from the liver of healthy wild-type (WT) and diet-induced obesity (DIO) mice with validation by biophysics methods. In total, 568 proteins were identified as potentially interacting proteins, and 296 proteins (52%) were present in both conditions. (**B**) Ranked list of Gene Ontology (GO) cellular components and (**C**) molecular functions of the identified proteins in both conditions. (**D**) Ranked list of Gene Ontology biological processes of the identified proteins in both conditions with hydrolase activity as the molecular function. *X*-axis shows gene count. Yellow star marks the inclusion of PON1 protein. (**E**) The interactome network of 37 proteins that are involved with phospholipid metabolic process, steroid metabolic process, and lipid transport, which are downstream of the molecular function of lipid metabolic process. PON1 is identified as the central protein involved in all three processes.

To identify the potential target proteins of vutiglabridin in a label-free and non-biased manner, the NPOT technique was used on liver tissue of both wild-type (WT) and high-fat-diet-induced obese (DIO) mice, as schematically shown in Figure 1A. The two different models were used to potentially separate targets in a healthy state versus a diseased state, and to strengthen the results of some proteins if they were found in both states. A total of 370 and 492 proteins were identified in the liver of WT and DIO mice, respectively, as part of the interactome with vutiglabridin, and 296 proteins were identified that were involved in both states (Figure 1A). To gain insight into the localization and functional roles of these proteins, we performed Gene Ontology (GO) analysis and found that the identified proteins are mostly localized to mitochondria, cytosol, and endoplasmic reticulum (ER), in this order (Figure 1B). The molecular functions of these proteins consisted largely of hydrolase activity, small molecule binding, and oxidoreductase activity, in this order (Figure 1C). From these data, the general processes mediated by these interacting proteins appeared to be related to either mitochondria and oxidoreductase activity, or the cytosol and hydrolase activity. The biological processes were specifically analyzed on the proteins that have hydrolase activity as their molecular function—since it represents a major catabolic function consistent with the reported effects of vutiglabridin—and showed lipid metabolic and fatty acid metabolic processes as the most significant processes affected (Figure 1D). Next, a protein–protein interaction network was created on the downstream biological processes of the lipid metabolism—phospholipid metabolic process, steroid metabolic process, and lipid transport—and among the 37 proteins, PON1 was identified as the central protein within the network. PON1 is known to mediate an enzymatic protection of LDL against oxidative modification and has been strongly implicated with the potential treatment of metabolic diseases including atherosclerosis and obesity [15]. Additionally, glabridin is reported to have direct molecular interaction with PON1 [36]. Therefore, PON1 was selected as a potential and promising investigative target of vutiglabridin, and this interaction was examined further.

### 3.2. Vutiglabridin Binds to rePON1 and Increases Its Enzyme Activity under Oxidative Stress Conditions

**Figure 2:**
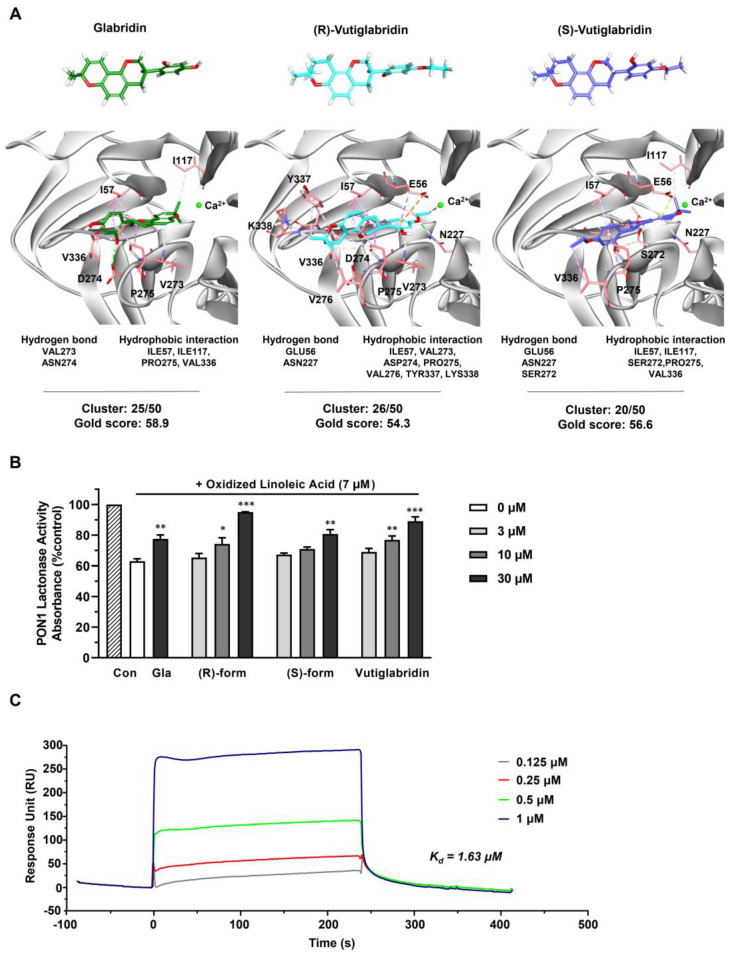
Vutiglabridin binds to rePON1 and increases it enzyme activity under oxidative stress conditions. (**A**) Chemical structures of glabridin, (R)-vutiglabridin, and (S)-vutiglabridin in stick model are shown in green, cyan, and purple, respectively. The oxygen and hydrogen atoms are in red and white, respectively. In the 3D structure of PON1 protein, the key residues of the binding site are represented by light red stick models. In the docked conformation of the three compounds, hydrogen bond, hydrophobic interaction, and unfavorable bump are shown as green, pink, and red dotted lines, respectively. The specific hydrogen bond and hydrophobic interaction sites, and the cluster analysis of compounds out of 50 trials and the GOLD fitness scores are written below each compound. (**B**) Glabridin (Gla), (R)-vutiglabridin, (S)-vutiglabridin, and vutiglabridin were incubated with rePON1 under oxidative stress conditions induced by oxidized linoleic acid at 7 μM. Lactonase activity was measured by spectrometry. Results are presented as the percentage of absorbance compared to the non-treated control (Con). The data are shown as mean ± SEM (*n* = 3). One-way ANOVA with Sidak’s multiple comparison test was performed. Statistically significant data are noted as * *p* < 0.05, ** *p* < 0.01, *** *p* < 0.001. (**C**) SPR analysis showing sensorgram of the interaction between vutiglabridin and rePON1 captured by anti-DDK/FLAG Tag antibody on the chip. The compound was diluted at the indicated concentrations. The sensorgram corresponds to normalized vutiglabridin signal (subtracted of reference surface and the buffer signal).

To investigate the potentially direct interaction between vutiglabridin and PON1, an in silico molecular docking simulation against PON1 was performed. The interaction between PON1 and glabridin have been previously studied in both in silico and in vitro settings, where glabridin was found to bind to PON1 mostly via hydrophobic interactions [32]. Therefore, we used glabridin as a positive control to confirm correct molecular docking and compare its binding interactions with those of vutiglabridin. The final docked conformations were selected based on cluster analysis and the GOLD fitness score. For glabridin, (R)-vutiglabridin, and (S)-vutiglabridin, respectively, cluster analysis showed consistent binding mode of 25 out of 50, 26 out of 50, and 20 out of 50, while the GOLD fitness scores were 58.9, 54.3, and 56.6 (Figure 2A). There was notable similarity in the sites of hydrophobic interactions between glabridin and vutiglabridin—Ile57, Ile117, Pro275, Val336—while the sites of hydrogen bonds were different. Overall, the in silico simulations showed that both enantiomers of vutiglabridin bind to PON1 in a manner comparable to glabridin.

Next, the effect of vutiglabridin binding to PON1 was investigated by measuring the enzyme activity of rePON1 under oxidative stress conditions. PON1 activity is reported to be decreased in oxidative stress conditions, specifically by oxidized linoleic acid (OX-LA) within the carotid atherosclerotic lesion lipid extract, with glabridin significantly protecting against such a decrease [36,37]. Therefore, we evaluated whether similar protective effects are induced by vutiglabridin. We incubated glabridin as a positive control at one dose (30 μM) and vutiglabridin and its enantiomers at three doses (3, 10, and 30 μM) for 30 min with rePON1 before adding OX-LA for 2 h and measuring the rePON1 lactonase activity. We found that both enantiomers of vutiglabridin significantly and dose-dependently protect against the decrease of rePON1 lactonase activity induced by OX-LA in a manner comparable to glabridin (Figure 2B). At 30 μM, under equivalent conditions, the rePON1 lactonase kinetic parameters K_m_ and V_max_ values were evaluated using iFIT program, which enables prompt and precise analysis of enzyme kinetic parameters [30]. We found that the catalytic efficiency (V_max_/K_m_) was non-significantly increased by vutiglabridin treatment compared to the OX-LA treated group (Table 1).

Lastly, to confirm that vutiglabridin binds specifically to PON1 and to find the degree of binding affinity, a surface plasmon resonance (SPR) analysis with rePON1 was performed, where the association and dissociation of vutiglabridin was measured with immobilized rePON1. Vutiglabridin showed clear dose-dependent association and dissociation with PON1, with a dissociation constant (K_d_) of 1.63 μM. Overall, these data show that vutiglabridin binds to rePON1 with relatively high affinity and protects rePON1 enzyme from oxidative stress-induced damage.

### 3.3. Vutiglabridin Increases PON1 Activity and Expression in the Plasma of WT Mice

**Figure 3:**
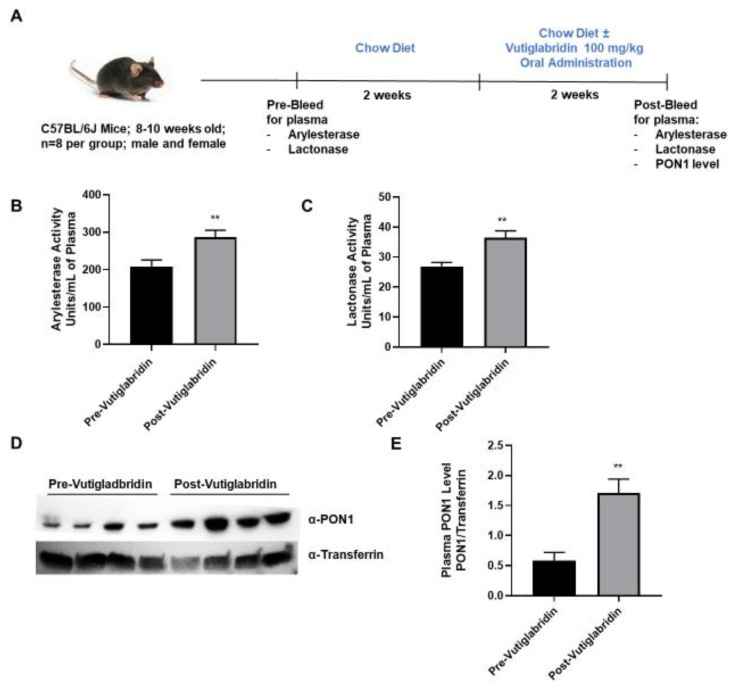
Role of vutiglabridin in modulating expression and activity of PON1. (**A**) Male and female WT, C57BL/6J mice between 8−10 weeks of age were pre-bled for plasma collection and were fed a chow diet for two weeks, followed by vutiglabridin at a dose of 100 mg/kg in their chow diet for another two weeks (*n* = 7–8/group), at which point the post-bleeding plasma collection occurred. Arylesterase (**B**) and lactonase (**C**) assays were performed on the plasma collected from the mice for comparison of pre- vs. post-vutiglabridin treatment (**D**). PON1 expression (*n* = 4/group) was evaluated in the plasma of male, WT mice via Western blot analysis, normalized to transferrin, and quantified as a ratio of PON1 to transferrin (**E**). The data are represented as mean ± SEM. Student’s *t*-test was performed. Statistically significant data are noted as ** *p* < 0.01.

In addition to the in silico and in vitro findings, the potential role that vutiglabridin might play in modulating PON1 expression and activity was assessed in WT C57BL/6J mice. As shown in Figure 3A, plasma from WT mice (*n* = 8 per group) was collected prior to the start of treatment as a baseline metric. The mice were allowed to recover for two weeks, and then vutiglabridin was orally administered at 100 mg/kg along with the chow diet for additional two weeks. PON1 arylesterase and lactonase activities were determined in the plasma collected pre- and post-vutiglabridin treatment. Both arylesterase and lactonase activities were significantly increased in the plasma from post-vutiglabridin treatment when compared to pre-vutiglabridin treatment (Figure 3B,C respectively).

Next, the protein expression of PON1 in the plasma was determined both pre- and post-vutiglabridin treatment. PON1 protein expression was significantly increased in the post-vutiglabridin group, when compared to pre-vutiglabridin controls (Figure 3D,E). Overall, in WT mice, vutiglabridin increased both PON1 enzymatic activity and protein expression level in the plasma, which is consistent with the in vitro findings that vutiglabridin binds to and protects PON1 protein from damage.

### 3.4. Vutiglabridin Protects against Diet-Induced Obesity in Hyperlipidemic LDLR^−/−^ Mice

**Figure 4:**
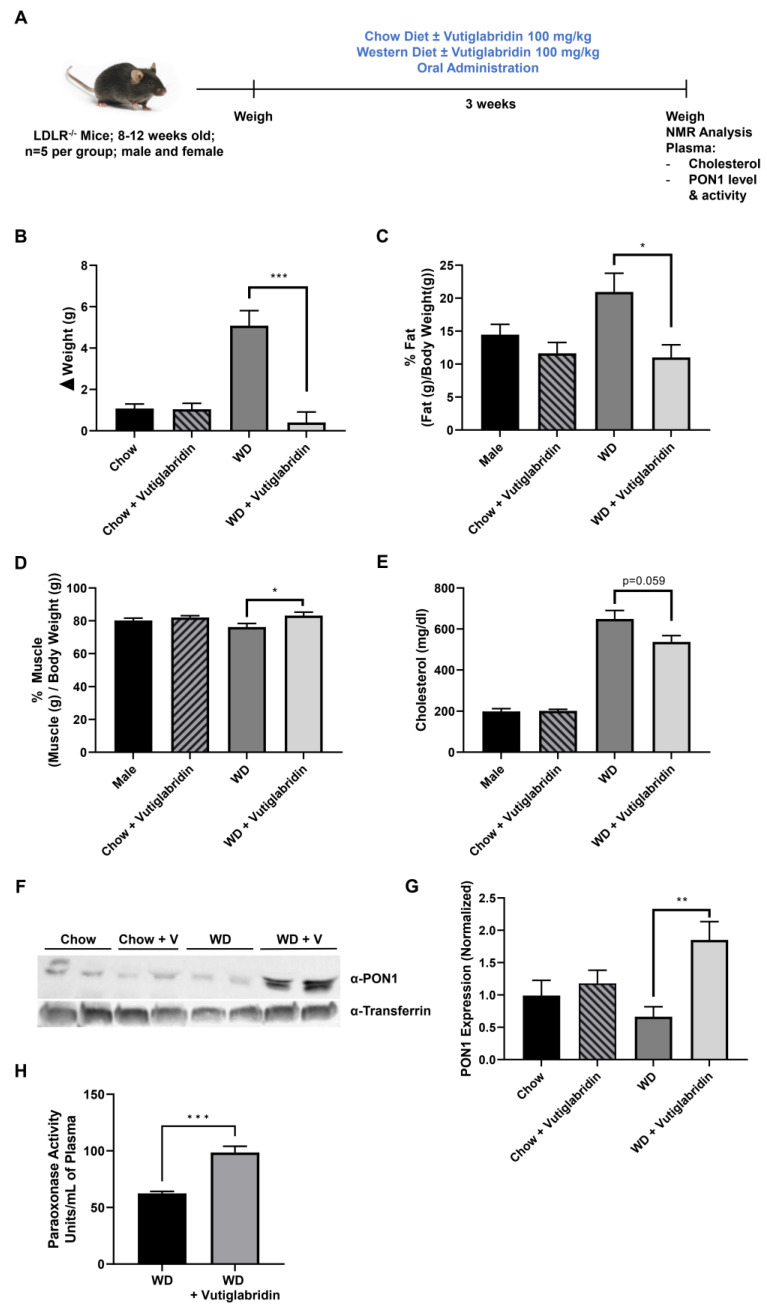
Vutiglabridin protects against diet-induced obesity in hyperlipidemic mice. (**A**) LDLR^−/−^ mice were utilized to investigate the role of vutiglabridin against diet-induced obesity and hyperlipidemia. Mice were fed four different diets for three weeks: Chow ± vutiglabridin or Western diet (WD) ± vutiglabridin. Dosing of vutiglabridin was at 100 mg/kg. Both male and female mice, between 8–12 weeks of age, with five mice per group were used. The mice were weighed prior to the start of the experiment. At the end of the third week of feeding, the experiment was terminated. (**B**) Changes in body weights were determined between the weight of the mice before the start of the experiment and final weights prior to termination. (**C**) Fat mass changes were assessed and quantified as percentage of final fat mass over total body weight × 100. (**D**) Muscle (lean) mass changes were assessed and quantified as percentage of final muscle mass over total body weight × 100. Plasma total cholesterol levels (**E**) were also determined. PON1 expression was evaluated at the end of the study in all groups via Western blot analysis, as reflected in the representative image (**F**). PON1 expression was normalized to transferrin and quantified as a ratio of PON1 to transferrin (**G**). The plasma paraoxonase activity as PON1 specific enzyme activity was measured in WD-fed groups (**H**). The data are represented as mean ± SEM. One-way ANOVA with Tukey’s multiple comparison test was performed. Student’s *t*-test was performed for (**H**). Statistically significant data are noted as * *p* < 0.05, ** *p* < 0.01, *** *p* < 0.001.

We sought to determine whether the protective properties of vutiglabridin can alleviate diet-induced obesity in hyperlipidemic LDLR^−/−^ mice, and whether these effects are at least in part mediated by PON1. Male and female LDLR^−/−^ mice (*n* = 5 per group) were fed a chow diet or a WD, high in fat and sugar, with or without vutiglabridin at 100 mg/kg for three weeks (Figure 4A). Body weights were measured at the beginning, weekly, and at the end of the study to evaluate weight changes. In chow diet-fed LDLR^−/−^ mice, there was approximately 1 g of weight gain within three weeks, and vutiglabridin administration did not induce any change in body weight gain. However, in WD-fed LDLR^−/−^ mice, vutiglabridin administration significantly reduced the body weight gain compared to its control (mean weight gain of 5.1 g vs. 0.4 g in non-treated vs. treated) (Figure 4B).

Next, using NMR, we determined the differences among the four groups in terms of fat and muscle (lean) mass, which are represented as a percent of fat/muscle mass over the total body weight. Similar to the pattern we observed in body weight change, vutiglabridin did not significantly change percent fat mass in chow diet-fed mice, but there was a significant drop in percent fat mass—from 21% to 11%, which is approximately a 48% reduction—in WD-fed mice treated with vutiglabridin compared to the non-treated control (Figure 4C). In addition, the percent muscle mass was significantly increased in the WD-fed mice treated with vutiglabridin compared to the non-treated control, while no statistically significant difference was observed in chow diet-fed mice groups (Figure 4D).

In addition, the total cholesterol level was evaluated in the plasma of the four groups. There was no difference in the cholesterol level in chow diet-fed mice groups with or without vutiglabridin; however, compared to those groups, WD-fed mice had significantly higher levels of cholesterol. There was a borderline significant decrease (*p* = 0.059) in WD-fed mice treated with vutiglabridin compared to the non-treated control (Figure 4E).

Lastly, in these obese and hyperlipidemic LDLR^−/−^ mice, the plasma protein expression and activity of PON1 were evaluated. Among the four groups, only WD-fed mice treated with vutiglabridin had a significant increase in PON1 protein level (Figure 4F,G). There was a significant and remarkable difference in PON1 paraoxonase enzyme activity between WD-fed mice treated with vutiglabridin compared to the non-treated control (Figure 4H). The protective effect of vutiglabridin on PON1 appears to have been amplified in the obese and hyperlipidemic mice, suggesting the strong therapeutic potential of vutiglabridin against metabolic diseases.

## 4. Discussion

In this study, we investigated the potential molecular target of vutiglabridin and identified PON1 as a direct interacting protein. Vutiglabridin binds to and protects rePON1 from oxidative stress in a manner like glabridin, and such effect is expectedly observed in the plasma of wild-type mice where vutiglabridin treatment increased PON1 expression and activity. Given these findings, vutiglabridin was tested in WD-fed LDLR^−/−^ mice as a model of obesity and hyperlipidemia where we observed a significant reduction of body weight, fat mass, and cholesterol, that was coupled with a remarkable increase in PON1 plasma expression and activity.

Unbiased target deconvolution of a small molecule is extremely challenging, because any type of labelling disrupts the chemical structure of the compound [38]. NPOT technique identified a total of 568 proteins that are either directly binding to vutiglabridin or interacting with those direct targets, using the liver proteins from WT and DIO mice. Through a series of pathway analysis, we identified PON1 as a central protein and confirmed the direct binding of vutiglabridin to rePON1. The strength of the NPOT technique lies in the non-label and non-biased aspect and the pathway analysis of the identified proteins which provide a macro-understanding of the localization and function of the protein interactome. However, the weakness is that each potential target protein cannot be compared against another in terms of binding affinity, unless otherwise tested in further analysis. Therefore, there are potentially other target proteins aside from PON1 that could contribute to the function of vutiglabridin. This paper focused solely on the cytosolic proteins with hydrolase activity as their molecular function; an equally or larger number of proteins were localized in mitochondria and mediated oxidoreductase activity, so future research will benefit from investigating the interactome of those proteins.

Vutiglabridin was found to bind to rePON1 with a relatively high affinity and protect its enzyme activity from OX-LA-induced damage. OX-LA was identified to specifically decrease rePON1 activity by oxidizing free sulfhydryl Cys284 and disrupting the protein stability. Glabridin was reported to bind to rePON1 at an allosteric and hydrophobic site that is different from the substrate binding site of the enzyme and modify the protein conformation to decrease the availability of Cys284, therefore protecting rePON1 from OX-LA [30]. Vutiglabridin and its enantiomers were found to bind to the same allosteric site and protect rePON1 from OX-LA-induced damage. Consequently, similar conformational change likely occurs with vutiglabridin treatment. In addition, treatment of vutiglabridin in wild-type, C57BL/6J mice significantly increased plasma PON1 level and enzyme activity. These data combined suggest that vutiglabridin, by binding to PON1, may enhance the protein stability of PON1 and/or protect PON1 from degradation in plasma. This hypothesis requires further investigation to determine whether the effects of vutiglabridin act to stabilize PON1 against natural degradation or degradation by oxidizing factors in the plasma of WT mice.

Oral administration of vutiglabridin at 100 mg/kg [21] in obese and hyperlipidemic LDLR^−/−^ mice significantly increased plasma PON1 level, while it decreased plasma cholesterol levels and total fat and body mass. This is a significant finding that supports the development of vutiglabridin for the treatment of both obesity and hyperlipidemia. The significant increase in PON1 protein level and activity induced by vutiglabridin treatment is consistent with the hypothesis that high-fat and high-sugar WD induces an oxidative environment that damages PON1, which vutiglabridin is able to mitigate by protecting PON1 against these oxidative conditions. However, this idea needs to be further validated by measuring PON1 turnover rate in WD-fed LDLR^−/−^ mice. In addition, the causative role of PON1 in mediating the beneficial effects of vutiglabridin was not examined in this study. A double knockout mouse of LDLR and PON1 may be constructed and used for such purpose. PON1 is a member of the paraoxonase family consisting of PON1, PON2, and PON3 [39]. PON3 is also a plasma HDL-associated protein that is reported to be markedly more efficient than PON1 in protecting LDL from oxidation [40], although the levels of PON3 are markedly less than PON1 [41]. Therefore, generating a total knockout of all the PON family may be needed to eliminate any compensatory effects from other PON2 or PON3. Regardless, in this study we observe a clear and consistent increase of PON1 levels and activity along with significant amelioration of obesity and hyperlipidemia with vutiglabridin treatment.

Overall, our results demonstrate that rePON1 is a direct interacting target of vutiglabridin, and that the modulation of PON1 by vutiglabridin may be used a potential therapeutic against obesity and hyperlipidemia.

## Figures and Tables

**Table 1:** Analysis of rePON1 enzyme kinetics of vutiglabridin under OX-LA stress conditions. The enzyme kinetic analysis of the recombinant PON1 was analyzed using a substrate, TBBL. Oxidized linoleic acid (OX-LA) and vutiglabridin were incubated in rePON1, and K_m_ and V_max_ values were extracted from the rePON1 kinetic graph using the iFIT program. V_max_/K_m_ was used as an index to see the catalytic function of the enzyme.

	PON1		
	-	OX-LA (7µM)	
		-	Vutiglabridin (30 µM)
K_m_ (µM)	155.78	102.11	134.52
V_max_ (µM/min)	92.34	46.71	69.14
V_max_/K_m_	0.59	0.46	0.51
Fold change of V_max_/K_m_	1	0.77	0.87

## Data Availability

Not applicable.
